# Dignity enhanced through faith & family support in palliative care: a qualitative study

**DOI:** 10.1186/s12904-024-01478-4

**Published:** 2024-06-07

**Authors:** Silva Dakessian Sailian, Yakubu Salifu, Nancy Preston

**Affiliations:** 1 American University of Beirut, Hariri School of Nursing, Riad El Solh, PO Box: 11 0236, Beirut, 1107, 2020 Lebanon; 2 International Observatory on End-of-Life Care, Division of Health Research, Faculty of Health and Medicine, Lancaster University, Sir John Fisher Drive, Lancaster, LA1 4YW UK

**Keywords:** Dignity, Palliative care, Qualitative research, Faith, Family, Middle East

## Abstract

**Background:**

Dignity is integral to palliative care. Illness can diminish it, causing hopelessness and the wish to hasten death. Yet, dignity is a complex multidimensional phenomenon, influenced by values and context. Understanding its varying interpretations can inform practice and policy. The aim of the study is to explore the understanding of dignity in adult patients with palliative care needs from a Lebanese perspective and how it is preserved during illness and while receiving health services.

**Design:**

Qualitative interview study underpinned with a social constructionist lens. Fourteen patients recruited from home-based hospice and outpatient clinics in Lebanon. Data analysed using reflexive thematic analysis.

**Results:**

Four themes were developed across all the interviews: (a) Dignity anchored through faith in God and religious practices; (b) Family support in maintaining physical, psychological wellbeing, and social connectedness; (c) Physical fitness, mental acuity, and healthy appearance through which patients may escape the stigma of disease, (d) accessible, equitable, and compassionate healthcare.

**Discussion:**

Dignity is elusive and difficult to define but faith and religious beliefs play a significant contribution in this study. For the participants, illness is seen as a natural part of life that does not necessarily diminish dignity, but it is the illness related changes that potentially affect dignity. Findings show the importance of family and children in preserving dignity during illness and how their active presence provide a sense of pride and identity. Participants aspired to restore physical, social, and mental well-being to reclaim their dignity and normalize their lives. Challenges related to physical appearance, memory loss, vitality, and social stigma associated with illness diminished dignity. Accessible, equitable and compassionate healthcare services are also crucial in preserving dignity. Participants valued clear communication, respect, and empathy from healthcare providers and identified affordability of care essential for maintaining dignity.

**Conclusion:**

Faith in God, and strong family ties are dominant elements to maintaining dignity in the Lebanese context. Relational connectedness with family, children or God is also a need in maintaining dignity in other communal countries with variations in emphasis. The study indicates that religious and cultural context shapes the needs and perceptions of dignity during illness. These findings are likely to be transferable to many Middle Eastern countries but also countries with strong religious and family ties globally.

## Introduction

Dignity is an integral principal in palliative care [1]. Patients with advanced chronic or terminal illnesses with palliative needs may experience heightened functional dependency, limited control, and loss of hope, that may negatively impact self-image, leading to isolation and poor quality of life [2–4]. The heavy burden of disease and increased vulnerability may even lead to a loss of dignity and a diminished desire to live in some individuals [1, 5]. Thus, preserving dignity, defined as “the quality or state of being worthy, honoured, or esteemed” or the personal perception of being respected by others and maintaining a good self-esteem [6], is crucial to enhance quality of life in palliative care [4].

Some patients have identified loss of personal privacy [7], physical and economic dependency [8] as threatening to dignity during illness. Health care providers’ attitudes and discourse while providing care, such as an authoritarian or curt communication, being omitted from decision making processes and lack of empathy can adversely impact a patient’s dignity [7–9]. On the other hand, respecting personal stories and patient autonomy has been found to uphold patient dignity [10].

The concept of patient dignity, measurements of dignity related distress, and dignity promoting interventions in palliative care have been well debated in the global north and some Asian countries showing variations in dignity interpretations [11–18]. However, dignity remains minimally assessed in the rest of the world including the Middle Eastern setting. Scarcity of knowledge from the Middle East leaves the utility of the existing research on dignity and its translation uncertain.

This paper aims to explore the understanding of dignity in adult patients with palliative care needs from a Lebanese perspective. Lebanon has endured a history of political and economic turmoil associated with sectarianism and an influx of refugees from neighboring countries [19]. People still die in uncomfortable and distressing situations often without access to palliation [20–22]. Lebanon is categorised as a group 3a country on the palliative care development scale where service delivery is isolated, heavily dependent on donors, with minimal availability of morphine [23]. As palliative care values permeate in countries like Lebanon, a deeper understanding of the key tenets of palliative care, such as dignity, is imperative to foster informed practices that enhance it. To provide culturally relevant care, it is important to ask what is the understanding of dignity for patients with palliative care needs from the viewpoint of the Lebanese?

To achieve the aim of this study, the following objectives were pursued:

A. Explore the understanding of the concept of dignity in patients with palliative needs as perceived by patients.
B. Examine how dignity is experienced while receiving health services in adult patients with palliative needs.

## Methods

### Study design

A qualitative study with patients experiencing advanced chronic illnesses or terminal conditions. Social constructionist theoretical lens underpinned the study. As such, knowledge is *interpreted and co-constructed* between the participants and the researcher and conceptualized in the prevailing socio-political and economic framework [24–27]. Reflexive thematic analysis was used [28, 29].The consolidated criteria for reporting qualitative studies (COREQ) guidelines was used to report the findings [30].

### Setting

The study took place in Beirut, Lebanon, where most large health care services are located. The setting was a home-based hospice service and an outpatient clinic for people with chronic healthcare conditions of a large tertiary private hospital. Both institutions serve patients from various socioeconomic and geographical backgrounds.

### Population

The participant inclusion criteria were the following:

- Adults of aged 18 years or more, since dignity conditions may vary with children [31].
- Living with advanced chronic or terminal conditions in need of palliative care such as solid or blood cancer, organ failures (heart, kidney, liver, lung disease), progressive neurologic/ autoimmune disorders (Parkinson’s, Crohn’s disease, Systemic Lupus Erythematosus), or other.
- Participants admitted to the hospital at least once in the past year, to ensure recent exposure to health care services.
- Participants who are cognitively intact, willing to be interviewed, capable of conversing in Arabic, English or Armenian and granting consent.

Those participants who were too ill or too distressed such as complaining of pain, breathlessness or other symptom that destabilizes their condition as identified by their physician or nurse were excluded. Participants who did not need palliative care, or had cognitive impairment were excluded, too.

### Sampling

Purposeful sampling of participants was implemented from whoever was accessible due to the restrictions imposed by Covid-19 [32].

### Recruitment

The physicians in the outpatient clinic and the nurses in the home-based hospice service, identified potential participants and facilitated access to the researcher (SDS) to contact them and provide details of the study. The researcher did not have any relationship with the participants. When recruitment became challenging due to the Covid-19 pandemic, additional participants were identified through snowballing, where a participant was identified through a previous participant. Time was allowed for questions or expressing concerns before consenting and data collection. The researcher SDS resides in Lebanon and has a background of nursing education and is currently a nursing instructor with good interviewing skills. The researchers (YS) and (NP) are experienced researchers in palliative care and were supervising the overall research process.

### Data collection

Written consent was obtained for the in-person interviews and verbally recorded for the telephone interviews. All participants who were approached accepted enrolment. Participants were asked about their understanding and experiences of dignity during their illness and while utilizing health care services. A topic guide, based on the findings of other empirical studies, (supplementary document), was designed to ensure the research question was covered [33, 34]. ‘What does dignity mean to you’, ‘How does it change during illness?’, ‘What actions enhance or diminish dignity while receiving health services?’ were some of the questions asked. Participants were encouraged to elaborate or clarify their responses when needed [35, 36]. As the researcher (SDS) was multilingual, the interviews were conducted in English, Arabic or Armenian at the participants’ request. Demographic information was collected before the interview (presented in Table 1). With the participants’ permission, interviews were audio recorded, and transcribed verbatim. Field notes about the setting, the patient’s mood, and overall tone were kept by the researcher after the completion of the interviews and included in the data analysis. Data collection stopped once information sufficiency had been achieved [37, 38].

### Data analysis

All interviews were translated into English. Some terminologies in Arabic or Armenian that did not have an accurate translation were kept in their original language in the transcripts to preserve their contextual meaning [39, 40]. The interviews were analysed using reflexive thematic analysis [41] following the six steps identified by Braun, Clarke [42]: (a) familiarizing with the data, (b) generating initial codes, (c) searching for themes, (d) reviewing the themes, (e) defining and naming the themes, and (f) producing the report [43]. The researcher remained loyal to the words used by the interviewees. The themes were developed inductively from the codes that appeared as patterns across the data. Analysis was conducted at the semantic explicit level as well as examining implicit meanings, concepts, assumptions and social implications [43]. For example, when a participant expressed concern that he is not able to take long walks or flirt with women, or when another one described feeling shameful when wearing a wig, these findings allowed the researcher to revisit the social concept of self-image and how it implicitly influences perceptions of dignity and self-worth. The process was reflexive and recursive requiring frequent revisions and refinements [41, 44–46]. NVivo qualitative data analysis software version 11 was used to manage the data, visualize connections to assist interpretation [47].

The credibility of the analysis was maintained through the researcher’s internal examination and awareness of personal biases and social positioning as a female nursing instructor that could have affected the findings. A reflexive diary was kept ensuring transparency throughout the phases of the research [42, 46]. Pseudonyms were applied to protect the identity of the participants.

As the research was conducted in Lebanon, and was part of a PhD thesis project, research ethics committee approval was secured from the American University of Beirut Institutional Review Board (IRB- ID: SBS 2020-0033) and from the Lancaster University Faculty of Health & Medicine Research Ethics Committee (FHMREC19139). Since the topic was sensitive A distress protocol was in place and the interviewees were offered to pause whenever needed to take time, reflect and then resume to avoid emotional distress or stop the interview if required [48].

## Findings

Fourteen patients were included in the study. All potential participants agreed to participate in the project. Four interviews were conducted in person in their homes (in line with Covid-19 guidelines) as per participants’ wishes, and the rest by telephone between September 2020 and April 2021. The interviews lasted between 5 min (this participant rapidly became too tired) to 79 min with a median of 38 min. Four themes and 15 sub-themes were developed in response to the research question (see Table 2). Each theme is described as to how it enhances dignity during advanced chronic and terminal illness in palliative care patients with illustrated quotes.

**Table 1:** Participant Demographics

Gender	Number of participants
Male	5
Female	9
**Religion**	
Christian	10
Muslim	4
**Marital Status**	
Married	11
Single	2
Widowed	1
**Age**	
45–54 55–64 65–74 75–84 85–94	3 1 6 3 1
**Diagnosis**	
Cancer	6
Organ Failure *(chronic obstructive lung disease; heart failure, kidney failure)*	6
Neurological disorders	2

**Table 2:** Themes and Supporting Subthemes

‘I have my faith you know… whatever will happen will happen.’	1. Dignity is a loose and abstract concept. 2. Dignity is inherent and not affected by illness. 3. Illness is accepted as a regular life event in older participants. 4. Dignity in illness is enhanced through faith.
‘Thank God I have my Children.’	1. Bearing children reinforces dignity. 2. Patients rely on children and spouses for support and personal care during illness. 3. Family highly involved in patient health care. 4. Visitors and staying connected boost dignity during illness.
‘I changed upside down…’	1. Maintaining energy to carry on with regular physical and social activities. 2. Preserving memory and cognitive ability. 3. Maintaining outer appearance, image, and social reputation. 4. Illness is stigmatized; hence, patient is eager to finding cure to preserve dignity.
‘The physician kissed me on the forehead and told me nothing is wrong with me.’	1. Compassionate care and presence of health care providers restores dignity. 2. Clear and non-judgmental communication from health care providers. 3. Accessible, affordable, and quality services and medicine for all patients regardless of social or economic rank.

### Theme one: dignity anchored through faith in God and religious practices. ‘I have my faith you know… whatever will happen will happen.’

In this theme the fluidity in understanding the nature of dignity was explored and how it is enhanced through faith, religious beliefs, and morality as viewed by the participants who came from a range of religions. Whenever patients were questioned about their perceptions of dignity, many did not comprehend the question and so the interviewer gave examples of life situations that may be dignifying such as weddings. Participant’s understanding or description of the concept of dignity was blurry regardless of gender, language, or ethnic group. Participants said that the word was loose and difficult to encapsulate. Others explained that dignity is something felt *within* the self, associated with emotions.

- *‘I don’t know “ezet nafs” (self-esteem) and dignity are so vague and can’t be defined and limited. It represents your inner being and world…. I do not know…. If you want, ask me more specific topics and I can answer.’ Patient # 5*

The concept of dignity was not necessarily related to health status, instead it was an inherently preserved human feature. Illness was regarded as a natural occurrence in life, something that anyone may experience without damaging their dignity or worth. Some participants believed that illness is predetermined from the creator, and that the almighty God is in control of everything, the source of offerings as well as losses.

- *‘No, no for me dignity is not affected by illness, they are not in the same boat…. Dignity for me has a different stance, different understanding. Illness is completely something else, why should it affect/ damage my dignity?’ Patient # 1*

Participants stated that dignity during illness is enhanced through faith and loyalty to the Almighty and religious practices. It is cultivated through demonstrating devotion to the religious virtues and values inscribed in sacred scripts of their religion, ‘*do not harm people, be good, do not gossip’ Zein*, establishing a closer connection between the individual and divinity. Participants expressed consolation and a safe refuge for their dignity through their faith in God who ‘listens to the sufferers’. Through prayer, watching religious television programs, reciting verses, and participating in WhatsApp spiritual group communities, participants evoked a sense of hope and wellbeing fostering their dignity.

- *‘It was not a personal or lonely struggle with the medical condition, but I felt that someone, the Lord, was with me… and the dign ity I received from my faith made me feel that someone is with me, I am not alone… there are things that cannot be fully expressed in words… you just feel them, you feel the presence of the Lord with us.’ Patient # 9*

### Theme two: family support in maintaining physical, psychological wellbeing, and social connectedness ‘Thank God I have my children.’

Participants explained that family members, children, and their social relationships are the building blocks and fundamental mesh where dignity is safeguarded during illness. The interviews reveal that living in or establishing a family and bearing children are core societal values that reinforce individual dignity, identity, and wellbeing. According to participants, being surrounded by family members and children is considered a loving shield, protective against physical, psychological, or economic threats to dignity. The family’s presence around the patient during hospitalisation is perceived as of utmost necessity as it feeds into the person’s identity, social status, worth, and dignity.

- *‘Dignity will change when the person does not have a family to dignify him/her and take care of him …, this is when dignity changes. However, thank God, I have my children, they work, and they are employees, so they preserve my dignity. Thank God’ Rima Patient # 3*

Children are regarded as the legacy of the participants to whom they pass on their values, ‘wisdom’, and personal stories, finding fulfillment and continuity that enhances dignity.

- *‘My wife and children are very good [hamdellah], and I live with them. My wife is also very good, she takes care of me … My children are with me, my wife is near me, and I own a house (an apartment) and the children do for me whatever I need, you understand?’ Patient # 4*

Married participants take pride in their children and those with no children celebrate their own personal successes, their achievements, and involvement in the community as their archive of personal dignity.

- *‘yes, I forget everything, but they (my children) are above all, they are the most important thing for me, especially that I have my condition, for me, my family is the most important thing’ Patient # 1*

A loving husband or a wife, who assists in the daily needs, and is loyal and empathetic towards them is a major source of support to boost dignity *‘the love that I enjoyed surrounding me was amazing ….’ Nelly*. In contrast, a husband, or a wife who is distant, uncaring, or not involved in the care reduces dignity and is even a source of distress.

- *‘even my husband didn’t take care of me, neglected me “[ma tallae feyye]” (he never laid eyes on me), I was trying to support my back and my lungs were hurting as if I am holding one ton of heaviness on my spine, and at the same time, I have to do my house chores, I needed to go up and wash the dishes and my tears filled my eye [dmooeei aala aaynayye]…So, I did not find any help from my husband ‘ Patient # 7*

Having visitors at times of illness and receiving calls is a sign that the individual is respected and missed in the social circle. Family members or friends living abroad come over to visit and support the ill person. This is a cultural norm that bears the meaning of reciprocity in caring, courtesy, and respect to the sick.

- *‘it’s not only that my brothers came over from Europe to visit me, I tell you all the whole neighbourhood came over for a visit. All the neighbourhood was here. If they don’t respect or care for me and I don’t respect them, do you think they would have come to see me?’ Patient # 2*

### Theme three: physical fitness, mental acuity, and maintaining healthy appearance through which patients may escape the stigma of disease ‘I changed upside down…’

Participants aspired towards restoring their physical fitness, mental and social wellbeing, vitality, and normal life. They hoped to escape from illness and its attached stigma, to reinstate normalcy, and restore dignity. Preserving physical energy, functionality, and appearance, were one of the defining elements of dignity in illness. The ability to move around, take care of personal needs independently (toileting, bathing, eating), be symptom free, pursue a career, go to outings for relaxation were other core elements of feeling dignified.

- *‘The things that changed in me are … what can I say…I changed upside down. I was a gentleman, I loved going to trips, to have fun, I love enjoyment and party ….my condition is not the same now it turned upside down. This is the truth…I used to walk from here (cheifat area) to Beirut governmental hospital and come back on my feet. It’s not the same now.’ Patient # 2*

Memory loss or mental confusion is mentioned as a barrier to maintaining dignity as it takes away not only the ability to make decisions, but also the capacity to start a meaningful conversation or socialise with others. As expressed by one of the participants, without her memory she was ‘*living in a trance or void’ Mary*, she was invisible like having lost her identity.

The outer appearance of the participant, such as maintaining intact skin, preserving the whole body without losing a body part, for example a breast, enhanced dignity. Clean clothes, healthy hair, teeth, were fundamental to self-esteem that boosted dignity particularly for women. Wearing a wig due to chemotherapy-induced alopecia was one of the most frustrating consequences of the treatment. Women tended to feel embarrassed, unable to discuss hair loss openly, coping with it through concealment and by wearing additional make-up.

- *‘Hmmm… I will tell you that for me the worst thing was my hair loss and the use of a wig. This was a topic that was very difficult for me to accept. Even when things were well, and I resumed going to work (as a teacher at school) that topic I couldn’t talk about … I was really touched by it and ashamed of ….’ Patient # 10*

On the other hand, men were mostly annoyed by their lack of physical fitness, fatigue, and limited energy.

Cancer was still regarded a stigma ‘*the condition’*, that is not talked about openly but is discussed through gossip or side conversations in the social circle. Pity expressed by society towards the patient elicited the perception of being weak, disempowered, and vulnerable. It reinforced demoralization that did not resonate with dignity.

- *‘Hmm…maybe…eh, when, for example, when you get sick, and people know that you are sick they start looking at you in a different way…eh…in a way that they pity you…eh. this is …I think this is not right… eh… they start …eh… labelling you. eh…which is …eh… which in my opinion is very wrong.’ Patient # 11*

### Theme four: accessible, equitable, compassionate, healthcare. ‘The physician kissed me on the forehead and told me nothing is wrong with me.’

Compassionate, quality, and affordable health services enhanced participants’ experiences of dignity and indifference, or limited access demoted it. This theme also included the need for clear communication, engaged physicians, respect for patient preferences, and equal access to quality health care. A kiss on the forehead in Arabic cultures is associated with acknowledging the grief of the other and aiming to provide comfort [49]. Health providers who approached participants with sensitivity and exhibited genuine caring, providing attention and hope for better wellbeing, were regarded as fostering their dignity.

- *‘The physician came to my room and asked about my wellbeing. I told him I had fever that day, he told me “come, come“, he kissed me on the forehead and told me “nothing is wrong with you”. He removed my leg stockings and told me to move my legs and start walking….’ Patient # 10*

Participants wanted respectful and a non-judgmental approach during their health service encounters expecting a physician who is competent and takes enough time to assess, listen and then propose a plan of care. Physicians who were available to answer phone calls, performed frequent ward rounds displaying an approachable attitude, and provided support to the patient during chemotherapy sessions, were regarded as ideal in fostering dignity. In contrast, physicians who remained distant in their posture or attitude, were volatile in their moods or non-empathetic in their responses, hurried in their communications were not favored.

- *‘For me it is important that a doctor is always a good listener and listens to all questions …This is what I want. These things comfort me. Sometimes they do not tell the truth the whole truth, though there are physicians who explain all the phases of the disease’ Patient # 13*

Information sharing was regarded to be an important aspect of care that enhanced dignity irrespective of language, gender, or age. Clear and simple information about side effects of treatment, chances of full recovery, the plan of care, upcoming procedures, was of utmost importance to all participants to safeguard dignity during health encounters. Hiding information or not fully disclosing all aspects of care was disappointing and diminishing to patient dignity. One participant was skeptical about the non-convincing explanations of a physician when he asked about potential complications of radiation therapy on his sexual health.

- *‘Once I told him ( the doctor) that my energy is low as a male, my energy is low, he told me till we finish the treatment, then you will be like a horse. But, when do we finish the treatment? Pause…. He had told me that this is my treatment for the rest of my life, is he deceiving me? or cheating on me or giving me a satisfying answer so that he is acceptable’ Patient # 2*

A variation in the quality of care or access to medicine was a concern for participants who could not afford to pay or were not politically affiliated. As most health services were privatized and expensive, the socioeconomic status or the power to afford services categorised participants as privileged, or less advantaged to access quality, timely, and expert care, or medicine.

- *‘At this hospital, outpatient health care depends on time spent with the physician, sometimes physicians don’t spend sufficient time with the patient, and this is not fair, and a very bad thing. If you have a long session, they charge a specific high fee, and if a short session they charge less’. Patient # 8*

These occurrences were interpreted as unjust, violating patient rights and diminishing the dignity of those who could not afford it.

- *‘If anyone belongs to a religious group, he is admitted to the hospital easily, Also, if you are a politician or something important, you will have no problem with the hospitals… it’s a big deal’. Patient # 11*

## Discussion

The study highlights that participants had a fluid understanding of dignity and struggled to define it, but they found that faith, religious beliefs, and moral values contributed to its enhancement. They believed that dignity is an inherent human feature, not dependent on health status. Illness was seen as a natural part of life and did not necessarily diminish dignity; participants suffered from the physical impact of illness it had on their life. The study also stresses the importance of family and children in preserving dignity during illness. Having a supportive caring family, especially children, reinforced dignity and provided a sense of pride and identity. Patients found a safe refuge in their families during their most vulnerable times that was often associated with a chain of losses. Being embraced and taken care of through family network restored personal dignity. Participants aspired to restore physical, social, and mental well-being to reclaim their dignity and normalize their lives. Challenges related to physical appearance, memory loss, vitality, and social stigma associated with illness diminished dignity. Finally, the study emphasizes the significance of compassionate and affordable healthcare services in preserving dignity. Participants valued clear communication, respect, and empathy from healthcare providers, and identified affordability and equal access to quality care essential for maintaining dignity.

The idea that dignity is elusive and difficult to define is aligned with other research findings [2, 8, 50]. Restoring dignity through faith in God and prayer can be seen as nurturing a sense of solace, reconciliation, and perseverance in some United States’ (US)and Chinese communities, too [51–54]. Religious rituals and traditions were major coping mechanisms of patients rooted in the daily routine upholding a sense of wellbeing and dignity. Patients longed for having peace with their creator and observing religious set of values which was a part of broader sense of spirituality but emphasized through commitment and faith in deity.

In Asian and African communities, dignity is at a dynamic interplay, often reciprocal, intertwined with the empathetic bonds and sense of connectedness [55] within the patient’s immediate family and their children [13, 15, 56]. In Western studies, the concept of individual autonomy and self- determination takes precedence [57, 58], whereas in collective societies, a familial approach to dignity is more prominent [55, 59, 60]. Participants in communal cultures such as Lebanon, perceive family and children as essential to securing their own dignity and personal legacy [60, 61]. Family members and children are often the primary caregivers, creating a safe space for healing and maintaining a sense of value in times of vulnerability [15, 56].

Across various regions including the U.S, Canada, Asia, and Europe, research participants consistently associate a positive sense of dignity with autonomy, the ability to carry out daily activities independently [62, 63], and communicate effectively with others [2, 58, 64–67]. However, the Lebanese culture, like the African, places a significant emphasis on outer appearance and physical fitness, assuming any decline in these aspects a threat to dignified social interactions [68, 69]. The stigma surrounding cancer compounds the distress, as physical changes diminish dignity even leading to non-adherence to therapy [70, 71]. It is relevant to state that this research has adopted the social constructivist paradigm where knowledge is co-constructed between the researcher and the participant and provides the unique perspective and voice of the Lebanese participants that could be useful in understanding similar contexts [27].

Patient dignity appears to be universally at risk when health providers fail to allocate sufficient time for listening to patient needs, or rush through their interactions [14, 72, 73]. Effective communication beyond the rigid professional boundaries, using simple and kind language seems to be a shared patient need in different parts of the world [72, 74, 75].

In Lebanon, the absence of universal health coverage creates a situation where access to quality healthcare becomes a privilege for the affluent or well-connected, burdens the less privileged subgroups financially and socially [76]. This issue is not unique to Lebanon but is also observed in other healthcare systems like the U.S., where socioeconomic wellbeing plays a crucial role in accessing dignified healthcare services [77]. The lack of equitable access to healthcare highlights the importance of addressing socioeconomic disparities and delivering equitable and inclusive care to uphold dignity in the face of illness and healthcare needs.

In this section the findings are compared against the Chochinov Model of Dignity [78] that comprises three main categories: (a) illness related issues, (b) dignity preserving repertoire, and (c) social dignity inventory. The themes of this study have an overall coherence with the categories of the Chochinov Model with some variations in emphasis or interpretation. The subthemes mentioned in the *Illness related issues* such as physical distress, maintaining physical functionality, cognitive wellbeing are also important elements in maintaining dignity in the Lebanese context. Nonetheless, two new sub-themes of dignity were identified in this study: (a) healthy outer appearance, and (b) accessible, equitable care that does not appear in the Chochinov model. Preserving a healthy physical appearance is a new theme key for preserving self-image and thereby avoiding stigma related to the illness. Most participants expressed that financial hardships and poverty limited access to needed resources indicating that economic wellbeing is a precondition to maintaining dignity. In this regard, *‘Accessible and equitable’ care* is crucial to preserve patient dignity while receiving health services, a theme missed in the Chochinov model. Inequalities and health disparities among citizens based on social class damages patient dignity as it restricts access to essential health services [79].

In addition, some of the sub-themes identified in the model hold a nuanced interpretation or have a different emphasis such as ‘social support’, ‘autonomy’, ‘role preservation’, ‘generalisability / and longevity’.

Faith and family support were the predominant coping practices through which patients invoked inner strength, (‘resilience’), and facilitated *‘*accepting the illness’. ‘Resilience’ and ‘accepting the illness’ are subthemes that existed in the model but without being related to religious faith or family support. Presence of children and spouses helped preserve dignity as they were seen as a source of ‘pride’ and ‘legacy for life’, again subthemes that existed in the *dignity preserving repertoire* category of the model but without being related with family support. ‘Role preservation’ in the traditional family hierarchy secured a sense of normality where patient ‘autonomy*’*, a subtheme in the model was replaced with a ‘relational autonomy’ among the family members. Due to the intense interdependence of the patient within the family fabric, individualistic autonomy did not surface rather there was collective decision making amongst the family members about health care issues in palliative care patients [80]. The theme of ‘c*are tenor’* mentioned in the model was also regarded an essential element of preserving dignity in this study where timely and clear information, and an authentic presence with the patient enhanced dignity [81]. The themes of being a ‘Burden to others’ and ‘Aftermath concerns’ mentioned was not identified a pattern across the data probably because patients believed it is the family members’ duty to care for the sick.

## Strengths & limitations

This research provides an initial understanding of the concept of dignity within palliative care from an Arab- Lebanese perspective where the topic is unresearched. It reveals the unique cultural understanding of dignity in patients with palliative care needs that may be useful to inform practice, care, health education and policy in Lebanon and nearby countries with similar geopolitical, and socioeconomic constructs.

Though telephone calls provided greater access to participants from remote areas and allowed participants to discuss sensitive issues whilst maintaining confidentiality and privacy [82, 83], participants’ actual home environment was sometimes missed. Also, participants were dominantly from a Christian community that may not reflect the religious dynamics of the country, or those who may not identify with any religion. The findings were affected by the dire socio-political and economic state of the country as well as the Covid-19 pandemic. Thus, it is possible should there have been economic and political stability the findings may have had a different emphasis. Implications of these findings for practice, policy and research are elaborated below.

### Implications for practice

The findings suggest that to enhance patient dignity, healthcare facilities should prioritise family presence and visits, provide space for spiritual practices, or spiritual referrals, and utilize digital applications to facilitate connecting with family and friends. Healthcare providers need to demonstrate active engagement with the patients and communicate full information about their choices of care and their implications. Healthcare team members and staff should receive education about practices that enhance dignity, tailoring interventions accordingly. Moreover, to foster a dignity-conserving culture, delivering compassionate and inclusive care is paramount especially for those from different socioeconomic backgrounds who may face barriers in accessing quality health services.

### Implications for policy

It is crucial that a national strategic plan for equal access to health care is planned and implemented based on the principles of equity and social justice that funds health care to all citizens. This could help reduce the social gap in accessing health services and promote dignity within palliative care and overall health.

### Implications for research

Exploring the perspective of health care providers regarding dignity and compare it for coherence with patients’ views is essential. Future studies that examine the root causes or interventions to foster equity, social justice and dignity in health care would be useful. Also, It would be interesting to pilot the patient dignity inventory-assessment tool [84] and examine how dignity therapy [85], or dignity conserving interventions [86] work to safeguard dignity in a Middle-Eastern culture.

## Conclusion

This study highlights the multifaceted nature of dignity among palliative care patients in Lebanon where familial, societal, and broader socio-political, religious factors shape its understanding [87–89]. Whilst conducted in Lebanon, the findings have implications to the Middle East and potentially wider global community where understanding cultural context is key in dignity-conserving care. Healthcare approaches that are mindful of the need of the patient to connect with their religious beliefs and practices, family, and children must be considered. Compassionate presence, clear communication and equitable access to care are fundamental aspects of the health care services that promote patient dignity in palliative care settings.

## Data Availability

The datasets generated and/or analysed during the current study are not publicly available due participant confidentiality and privacy but are available from the corresponding author on reasonable request.
